# Supplementary material for: Comprehensive Evaluation of 17 Qualities of 84 Types of Rice Based on Principal Component Analysis
Source: Foods. 2021 Nov 22;10(11):2883. doi: 10.3390/foods10112883 (PMC8622839; doi:10.3390/foods10112883)
Supplement: Supplementary file 1 [file foods-10-02883-s001.zip › Table S1.pdf]

Table S1 Comprehensive evaluation score of rice quality

Type	Score	Rank	Type	Score	Rank
0N-Tianyuanxiangjing	-0.38	49	350N-Yongyou4949	-1.35	69
25N-Tianyuanxiangjing	-0.71	60	0N-Yongyou7850	2.09	5
50N-Tianyuanxiangjing	-1.62	76	0N-Yongyou4949	1.93	6
75N-Tianyuanxiangjing	-1.60	74	25N-Yongyou4949	1.02	17
100N-Tianyuanxiangjing	-1.75	79	50N-Yongyou4949	0.79	24
125N-Tianyuanxiangjing	-2.03	83	0N-Meixiangzhan ||	3.03	1
150N-Tianyuanxiangjing	-1.75	78	25N-Meixiangzhan ||	1.65	10
175N-Tianyuanxiangjing	-1.56	72	50N-Meixiangzhan ||	1.46	12
200N-Tianyuanxiangjing	-2.26	84	100N-Meixiangzhan ||	0.86	23
250N-Tianyuanxiangjing	-1.76	80	125N-Meixiangzhan ||	0.44	31
300N-Tianyuanxiangjing	-1.55	71	150N-Meixiangzhan ||	0.70	25
350N-Tianyuanxiangjing	-1.25	66	175N-Meixiangzhan ||	0.94	19
25N-Jiafengyou ||	-1.31	68	0N-Lvyinzhan	2.82	2
50N-Jiafengyou ||	-0.59	58	25N-Lvyinzhan	1.45	13
75N-Jiafengyou ||	-1.17	63	50N-Lvyinzhan	1.83	8
100N-Jiafengyou ||	-1.20	64	75N-Lvyinzhan	1.42	14
125N-Jiafengyou ||	-1.70	77	100N-Lvyinzhan	1.66	9
150N-Jiafengyou ||	-1.77	81	150N-Lvyinzhan	0.19	38
175N-Jiafengyou ||	-1.62	75	175N-Lvyinzhan	0.87	22
200N-Jiafengyou ||	-1.56	73	0N-Huanghuazhan	2.78	3

250N-Jiafengyou II	-1.49	70	25N-Huanghuazhan	2.13	4
300N-Jiafengyou II	-1.27	67	50N-Huanghuazhan	1.86	7
350N-Jiafengyou II	-2.02	82	100N-Huanghuazhan	1.08	16
25N-Yongyou7850	0.90	21	150N-Huanghuazhan	0.58	28
50N-Yongyou7850	1.33	15	175N-Huanghuazhan	1.50	11
75N-Yongyou7850	0.97	18	0N-Jiafengyou II	0.30	35
100N-Yongyou7850	0.91	20	75N-Meixiangzhan II	0.19	39
125N-Yongyou7850	0.35	33	200N-Meixiangzhan II	-0.52	55
150N-Yongyou7850	0.67	26	250N-Meixiangzhan II	-0.40	51
175N-Yongyou7850	0.67	27	300N-Meixiangzhan II	-0.54	56
200N-Yongyou7850	0.09	42	350N-Meixiangzhan II	-0.89	61
250N-Yongyou7850	-0.08	43	125N-Lvyinzhan	0.17	40
300N-Yongyou7850	0.54	29	200N-Lvyinzhan	-0.54	57
350N-Yongyou7850	-0.26	47	250N-Lvyinzhan	-0.60	59
75N-Yongyou4949	-0.17	45	300N-Lvyinzhan	0.29	36
100N-Yongyou4949	0.54	30	350N-Lvyinzhan	0.16	41
125N-Yongyou4949	-0.47	54	75N-Huanghuazhan	0.32	34
150N-Yongyou4949	-0.40	50	125N-Huanghuazhan	0.36	32
175N-Yongyou4949	-0.10	44	200N-Huanghuazhan	0.21	37
200N-Yongyou4949	-1.24	65	250N-Huanghuazhan	-0.19	46
250N-Yongyou4949	-1.13	62	300N-Huanghuazhan	-0.47	53
300N-Yongyou4949	-0.32	48	350N-Huanghuazhan	-0.47	52
